# Investigation of commercially available recombinant and conventional *β*-glucuronidases to evaluate the hydrolysis efficiencies against *O*-glucuronides and *N*-glucuronides in urinary drug screening

**DOI:** 10.1007/s11419-025-00715-6

**Published:** 2025-03-05

**Authors:** Akira Namera, Takeshi Saito, Masataka Nagao

**Affiliations:** 1 Department of Forensic Medicine, Graduate School of Biomedical and Health Sciences, Hiroshima University, 1-2-3 Kasumi, Minami-Ku, Hiroshima, 734-8551 Japan; 2 Department of Emergency and Critical Care Medicine, Tokai University School of Medicine, 143 Shimokasuya, Kanagawa, 259-1193 Japan

**Keywords:** Recombinant *β*-glucuronidase, Hydrolysis efficiency, *O*-Glucuronides, *N*-Glucuronides, Drug screening

## Abstract

**Purpose:**

To achieve the rapid analysis of drug metabolites in urine, we examined the differences in the hydrolysis efficiencies against *O*-glucuronide and *N*-glucuronide by two commercially available glucuronidases and three commercially available recombinant ones.

**Methods:**

The metabolites analyzed included oxazepam-*O*-glucuronide, amitriptyline-*N*-glucuronide, and diphenhydramine-*N*-glucuronide. Hydrolysis was performed using commercially available five enzymes at two different temperatures, and the reaction progress was monitored for up to 360 min. The amount of hydrolyzed product was quantified using liquid chromatography–tandem mass spectrometry.

**Results:**

Although no enzyme selectivity was observed for the hydrolysis of *O*-glucuronide, the hydrolysis efficiency against *N*-glucuronide varied significantly, depending on the enzyme and reaction temperature. Among the enzymes evaluated, IMCSzyme 3S and the enzyme derived from *E. coli* demonstrated superior hydrolysis of *N*-glucuronides under optimal conditions. For IMCS RT, good results were also obtained by adding twice the amount of enzyme specified.

**Conclusions:**

Suitable enzymes and hydrolysis conditions were determined for the rapid and systematic screening of drug metabolites in human urine. These findings are expected to streamline the analytical workflow and reduce the need for tedious sample preprocessing.

**Supplementary Information:**

The online version contains supplementary material available at 10.1007/s11419-025-00715-6.

## Introduction

The abuse and misuse of various drugs remain a global concern and are regulated by each country. In addition to legally controlled drugs, overdose and abuse of over-the-counter drugs have recently emerged as serious social problems [1–4]. Moreover, it is often difficult to identify drugs consumed by patients admitted to the intensive care unit. In forensic autopsies, identifying drugs consumed by deceased individuals is even more difficult. In clinical cases, drug identification is guided by a toxidrome; however, in most cases, it is impossible to identify them. In forensic cases, typical clinical symptoms are usually not observed with a significant time lapse after death. Therefore, it is even more difficult to identify the type of drugs used. Qualitative analysis of drugs using rapid screening is useful for properly treating patients with intoxication in emergency medicine and estimating the cause of death in forensic cases. Urine is the most widely used specimen for drug exposure analysis owing to the extended drug detection window and its higher drug concentrations compared to those in the blood. In addition, urine can be collected noninvasively and conveniently during clinical emergencies [5]. However, limitations exist, such as the lack of correlation between clinical symptoms and drug concentrations in urine as well as the low volume of urine collected for analysis. Although there are numerous simple detection kits available in the market that can estimate the presence of drugs in urine, they test only for drugs, such as stimulants, opiates, and cannabis, which are legally regulated. Recently, the number of drugs that are undetectable using simple drug tests has increased, and there is an urgent need to resolve this issue. To address these issues, systematic analytical screening using chromatography coupled with mass spectrometry can be used for wide-range screening.

Notably, oxidation or reduction of a drug occurs after absorption into the body and it is eventually excreted in the urine as glucuronide or sulfate conjugates [6, 7]. Recently, new methods have been developed for the direct liquid chromatography–tandem mass spectrometry (LC–MS/MS) analysis of drug glucuronide and sulfate conjugates [8–10]. However, the unavailability of pure and standard glucuronides and sulfates for each drug makes it difficult to directly analyze and quantify the metabolites in urine. In these cases, glucuronides and sulfates are hydrolyzed under alkaline or acidic conditions, or by enzymes. In benzodiazepines, original drugs could not be identified by acidic hydrolysis because benzodiazepines are converted to benzophenones under acidic conditions [11, 12]. Therefore, enzymatic hydrolysis is typically used to degrade glucuronide [13, 14]. Conversely, the hydrolysis efficiencies of codeine and dihydrocodeine by enzymes are low [15–17]. Acid hydrolysis is typically used to cleave morphinan glucuronides [15, 18, 19]. In tetrahydrocannabinol, combined hydrolysis under alkaline conditions and an enzyme addition is performed for highly efficient hydrolysis [20–22]. Although such *O*-glucuronide conjugates are the focus of attention as urinary metabolites, certain basic drugs, such as histamine H1 receptor antagonists and antidepressants, cause excretion of substantial amounts of *N*-glucuronide conjugates in the urine [23–26]. Therefore, it is crucial to investigate the hydrolysis efficiencies against these *N*-glucuronide conjugates.

It is necessary to select an appropriate hydrolysis method depending on the target drug. However, it is difficult to estimate the drug content in each case before analysis using analytical instruments, and using a single hydrolysis method for unknown drug screening could prove useful. *β*-Glucuronidases are primarily extracted from various animals and bacteria and have been used for over half a century in the life sciences and diagnostics industries. Enzymatic hydrolysis is usually performed overnight at 37 °C [13, 14, 27] or for several hours at high temperatures according to the manufacturer’s instructions [28, 29]. The optimization of hydrolysis conditions using glucuronidase has been reported in forensic toxicology [30–32]. Notably, recently, recombinant enzymes capable of efficiently hydrolyzing glucuronide conjugates within short reaction times have been developed and products of uniform quality are commercially available. Recombinant enzymes have been used to hydrolyze morphine, cannabis metabolites, and other drug’s metabolites [17, 33–36]. The efficient hydrolysis of glucuronide conjugates in urine within a short time can significantly reduce the time of drug screening. In this study, we used this recombinant enzyme and several conventional enzymes that have been previously used for enzymatic hydrolysis to examine the time profile of hydrolysis efficiency based on the differences in each enzyme.

## Materials and methods

### Materials and reagents

Oxazepam-*O*-glucuronide, amitriptyline-*N*-glucuronide, and diphenhydramine-*N*-glucuronide were purchased from Cerilliant (Round Rock, TX, USA). Oxazepam, amitriptyline, diphenhydramine, and diazepam-*d*_*5*_ (internal standard: IS) were purchased from Sigma-Aldrich (St. Louis, MO, USA). Genetically modified *β*-glucuronidase IMCSzyme RT (> 200,000 units/mL), IMCSzyme 3S (> 50,000 units/mL), and rapid hydrolysis buffer were purchased from Integrated Micro-Chromatography Systems (Irmo, SC, USA). Fast *β*-glucuronidase (derived from *Patella vulgata*, 300,000–400,000 units/mL, aqueous solution) was obtained from Sigma-Aldrich. As a conventional enzyme, *β*-glucuronidase from *Escherichia coli* (1,000,000 units/g protein, lyophilized powder) and *Helix pomatia* (Type H-2, 85,000 units/mL, aqueous solution) were obtained from Sigma-Aldrich. Captiva EMR-lipid cartridge (1 mL, 40 mg) was purchased from Agilent Technologies (Santa Clara, CA, USA). Other chemicals were of special or LC–MS grade and were purchased from FUJIFILM Wako Pure Chemical Corporation (Osaka, Japan). Certified drug-free human urine samples were obtained from Clinical Trials Laboratory Services (London, UK). All drug stock solutions were prepared at a free concentration of 1.0 mg/mL using acetonitrile and diluted with acetonitrile as required for the experiments.

### Hydrolysis procedure

According to the manufacturer’s recommendations, the optimal hydrolysis conditions for each enzyme used in this study are listed in Table 1. The following proprietary buffers were used for hydrolysis of each *β*-glucuronidase: sodium acetate buffer (2 M, pH 4.8) for *H. pomatia*, phosphate buffer (2 M, pH 6.8) for *E. coli*, and sodium acetate buffer (2 M, pH 4.6) for Fast *β*-glucuronidase. Buffer was added at a ratio of 30 µL per 100 µL of urine. For IMCSzyme, the accompanying buffer was added in the amount specified in the instruction manual.

**Table 1:** Source of enzymes used in experiments and optimal reaction conditions described in instruction manual

Enzyme	Origin	Manufacture	Optimal pH	Optimal temperature (°C)	Optimal reaction time (min)
IMCSzyme RT		IMCS	5.5–6.5	20–25	15
IMCSzyme 3S		IMCS	6.0–8.0	55	30
Fast *β*-glucuronidase (recombinant)	*P. vulgata*	Merck	3.8–5.0	60–70	30
*β*-Glucuronidase (Type IX-A)	*E. coli*	Merck	6.0–7.0	37	
*β*-Glucuronidase (Type H-2)	*H. pomatia*	Merck	4.5–5.0	37	

Initially, the hydrolysis efficiencies of the five β-glucuronidases were assessed at two different temperatures (37 and 55 °C) for incubation periods of 15, 30, 45, 60, 120, 180, and 360 min with mixed urine of glucuronide of oxazepam, amitriptyline, and diphenhydramine (2 μg/mL final concentration in urine), using an aluminum block heater (DTU-1C; TAITEC, Saitama, Japan). However, the low-temperature incubation of IMCSzyme RT was done at room temperature (25 °C), and the high-temperature incubation of Fast *β*-glucuronidase was at 70 °C as recommended in the instruction manual. To compare the hydrolysis efficiency, the amount of enzyme added was set to be 2000 units/sample, the same just of the typical condition used for IMCSyme RT hydrolysis. Following hydrolysis, 5 µL of diazepam-*d*_*5*_ (10 µg/mL) and 500 μL of cold acetonitrile (− 20 °C) were mixed with the hydrolyzed urine samples, which were subsequently passed through a Captiva EMR-lipid for enzyme removal and sample cleanup. The filtrate was evaporated under a stream of nitrogen and the residue was dissolved in 100 µL of 30% aqueous methanol. The final solution was transferred to a sample vial for LC–MS/MS analysis. The hydrolysis efficiency was calculated as follows: % hydrolysis = A/B × 100%. “A” in the equation is the mean (*n* = 3) of the product peak area ratio against IS from glucuronide in the hydrolysis condition, whereas “B” is the ratio theoretically obtained as complete hydrolysis.

Second, IMCSzyme RT was used to evaluate the hydrolysis efficiency by varying the amount of enzyme and buffer. The enzyme and buffer were added in amounts 1–3 times higher than the amount recommended by the manufacturer. The subsequent hydrolysis and extraction steps were consistent with those described above.

### LC–MS/MS systems and conditions

The samples were analyzed using LC–MS/MS (Agilent Technologies 1200 liquid chromatograph-6420 quadrupole mass spectrometer). Separation was achieved by a ZORBAX Eclipse Plus C18 column (100 mm × 2.1 mm i.d., particle size of 3 μm) (Agilent Technologies). Mobile phases comprised 0.1% formic acid in water for solution “A” and 0.1% formic acid in acetonitrile for solution “B.” For benzodiazepines analysis, isocratic mode was used with the elution solvent of 50% B (v/v). For the separation of metabolites of etizolam and triazolam, InertSustain Phenyl (100 mm × 2.1 mm i.d., particle size of 3 μm) (GL sciences Inc., Saitama, Japan) was used with the elution solvent of 60% B (v/v). The flow rate was set to 0.2 mL/min. The peak area ratios of the analytes were obtained using multiple reaction monitoring (Table S1).

## Results

### Hydrolysis of *O*-glucuronide

**Fig. 1:**
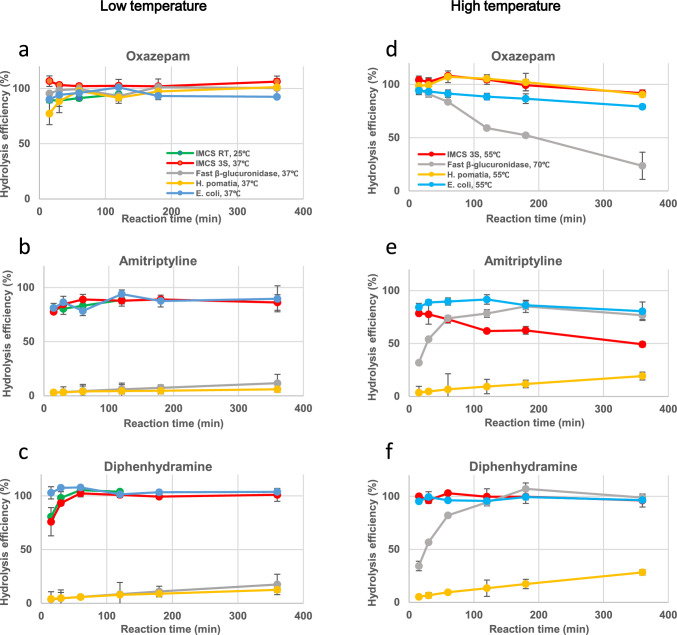
Time profiles of hydrolysis efficiency of each glucuronide using different originated enzymes

Oxazepam-*O*-glucuronide was efficiently hydrolyzed by all enzymes under low-temperature incubation within a short reaction time (Fig. 1a). Notably, the hydrolysis efficiency remained constant, throughout 360 min of reaction under these incubations. Even under high-temperature incubation, oxazepam-*O*-glucuronide was efficiently hydrolyzed within a short duration (Fig. 1d). However, when the enzymes derived from *E. coli* and Fast *β*-glucuronidase were used, the hydrolysis efficiency decreased, depending on the reaction time. The hydrolysis efficiency of E. coli decreased to 80% after 360 min, and the hydrolysis efficiency of Fast *β*-glucuronidase decreased substantially to 20% after 360 min.

To elucidate the cause of the reduced hydrolysis efficiency by Fast *β*-glucuronidase at high temperatures and prolonged incubation, oxazepam-*O*-glucuronide and the hydrolysis product, oxazepam, were separately added to the buffer and their stabilities were monitored. The oxazepam content decreased with increasing incubation time, and the results confirmed that oxazepam was unstable at high temperatures [37].

### Hydrolysis of *N*-glucuronide

The hydrolysis profiles of amitriptyline-*N*-glucuronide and diphenhydramine-*N*-glucuronide were similar at low-temperature incubation (Fig. 1b and c). Amitriptyline-*N*-glucuronide and diphenhydramine-*N*-glucuronide were efficiently hydrolyzed at low temperatures within a short incubation, except for *H. pomatia* and Fast *β*-glucuronidase (Fig. 1b and c). The hydrolysis efficiency remained constant even after 360 min of reaction. When both the enzymes derived from *H. pomatia* and Fast *β*-glucuronidase were used at low temperatures, the hydrolysis efficiency was very low. It failed to reach 20%, throughout 360 min of reaction.

At high temperatures, amitriptyline-*N*-glucuronide and diphenhydramine-*N*-glucuronide were efficiently hydrolyzed by *E. coli* and IMCSzyme 3S within a short incubation (Fig. 1e and f). On the other hand, the hydrolysis efficiency against amitriptyline-*N*-glucuronide and diphenhydramine-*N*-glucuronide by Fast *β*-glucuronidase increased gradually depending on the reaction time and reached 80% after 60 min. The hydrolysis efficiency against amitriptyline-*N*-glucuronide and diphenhydramine-*N*-glucuronide by *H. pomatia* increased slightly depending on the reaction time but did not reach 20% after 360 min. Although the hydrolysis of amitriptyline-*N*-glucuronide was achieved at high temperatures in a short incubation using IMCSzyme 3S, the hydrolysis efficiency decreased gradually depending on the reaction time, and that decreased to 50% after 360 min. To confirm that the hydrolysis efficiency of IMCSzyme 3S decreased when reacted at high temperatures for a long incubation period, the stabilities of amitriptyline-*N*-glucuronide and the hydrolysis product, amitriptyline, were monitored for the buffer solution without enzyme. The amitriptyline content significantly decreased, confirming that amitriptyline was unstable in the buffer at high temperatures.

Based on these results, the use of IMCSzyme 3S or *β*-glucuronidase from *E. coli* is recommended to enable high-throughput analysis of drug metabolites in human urine when the target is unknown.

### Effects of amounts of enzyme and buffer

**Fig. 2:**
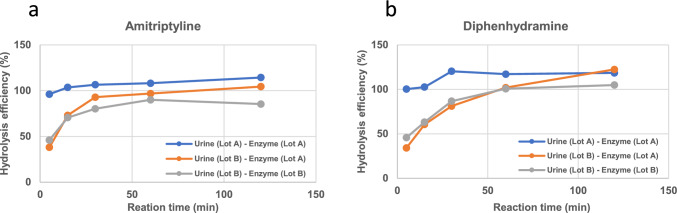
Effect of different lots of urine used on hydrolysis efficiency against *N*-glucuronide using IMCSzyme RT

When *N*-glucuronides were hydrolyzed by IMCS RT, hydrolysis efficiency varied depending on the lot of urine tested (Fig. 2a and b). However, this variability was not observed for *O*-glucuronide. When the hydrolysis efficiency against *N*-glucuronides was confirmed using different lots of enzyme and urine, it was suspected that the cause was the urine used. Notably, hydrolysis efficiency can be improved by increasing the hydrolysis time.

**Fig. 3:**
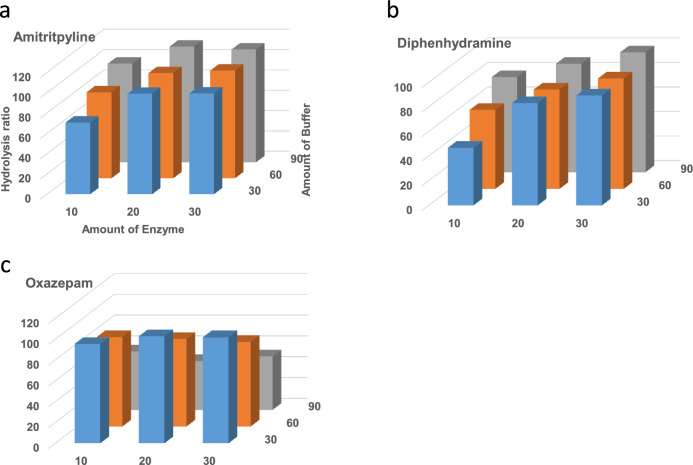
Effect of enzyme (IMCSzyme RT) and buffer amounts on the hydrolysis efficiency of each glucuronide

We investigated the amounts of enzyme and buffer required to minimize the hydrolysis time. The hydrolysis efficiency of the *N*-conjugate increased, depending on the amounts of enzyme and buffer added (Fig. 3a and b). By contrast, *O*-conjugates exhibited decreased hydrolysis efficiency, depending on the amounts of enzyme and buffer added (Fig. 3c). Considering these results and the cost of the enzyme, the amount of enzyme added was selected to be 20 µL for the hydrolysis of IMCSzyme RT.

### Application

**Fig. 4:**
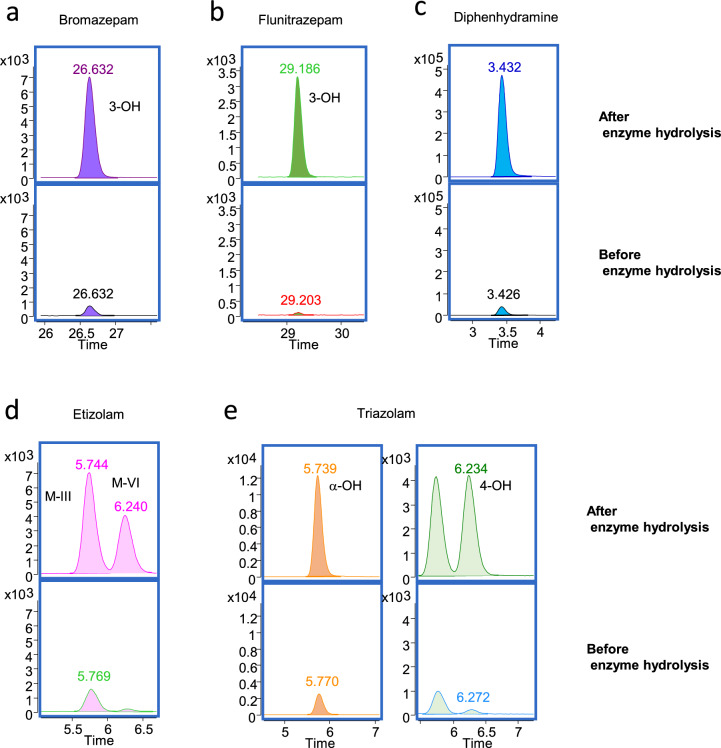
Change in peak areas of various *O*-glucuronides and *N*-glucuronides after enzymatic treatment (IMCSzyme 3S) of urine from persons suspected of ingesting drugs. Monitoring ions: 3-hydroxybromazepam; *m/z* 332.0 − > 78.1, 3-hydroxyflunitrazepam; *m/z* 330.1 − > 283.9, etizolam M-III, M-IV, 4-hydroxytriazolam; *m/z* 359.1 − > 340.9, α-hydroxytriazolam; *m/z* 359.1 − > 330.1

The recommended hydrolysis method by IMCSzyme 3S was applied to forensic autopsy cases where the intake of benzodiazepines or diphenhydramine was suspected (Fig. 4). Similar to the findings for oxazepam in this study, the concentration of the hydroxylated metabolite of the intake benzodiazepines became higher after hydrolysis than before hydrolysis (Fig. 4a and b). As long as the LC separation conditions are suitable, the separation of the metabolites of etizolam and triazolam is feasible, even when they are taken simultaneously (Fig. 4d and e). Similarly, diphenhydramine was detected at a higher concentration than the parent drug (Fig. 4c). The method proposed in this study offers a rapid and efficient method for drug screening in real forensic cases.

## Discussion

The optimization of hydrolysis conditions is critical to ensure complete deconjugation. The parameters including the amount of enzyme, incubation temperature, duration of incubation, and pH of the buffer influence enzyme hydrolysis. The enzyme activity must be sufficient to achieve complete hydrolysis of the conjugates present in the sample. However, the required amount of enzyme depends on the type and concentration of the target analyte as well as other hydrolysis conditions. In addition, enzyme selectivity and reactivity can vary based on the source and batch because these enzymes are prepared from natural products [16]. Due to the poor enzymatic hydrolysis efficiencies against morphine-6-glucuronide and codeine-6-glucuronide, the use of acid hydrolysis is required [15]. Moreover, there is another problem that the structural conversion of oxycodone to oxymorphone and codeine to morphine during acid hydrolysis has also been reported. Recently, recombinant enzymes were reported to solve these problems [38, 39]. The advances in biotechnology have enabled the production of enzymes with consistent quality using recombinant techniques. Therefore, enzymes with minimal lot-to-lot variation are now commercially available. Notably, cannabis components can be efficiently hydrolyzed using recombinant enzymes, which are expected to be useful [34]. In this study, we investigated the hydrolysis efficiency against *N*-glucuronide conjugates, which has rarely been reported, and examined the possibility of analyzing the conjugates for a short duration.

Conventionally, it takes several hours or overnight to hydrolyze *O*-conjugates, as reported previously [13, 14, 27–29]. However, our findings demonstrated that hydrolysis could be completed within 15 to 30 min, irrespective of the enzyme type used, although a higher enzyme concentration was added compared to previous reports. The specificity of the enzyme used for hydrolyzing the *O*-conjugate could not be confirmed, suggesting that the enzyme had a low selectivity for hydrolyzing *O*-glucuronide. Moreover, it can be hydrolyzed within a short duration without restricting the reaction temperature, except for Fast *β*-glucuronidase. According to the instruction manual for Fast *β*-glucuronidase, the optimal temperature for this enzyme is 70 °C. However, the hydrolysis efficiency against oxazepam-*O*-glucuronide decreased over a prolonged reaction owing to the low stability of the hydrolyzed product oxazepam. Consequently, the hydrolysis product oxazepam decomposed over a prolonged duration under these reaction conditions [37]. Therefore, when using Fast *β*-glucuronidase, it is necessary to strictly set up the reaction time and complete the hydrolysis reaction in a short duration to suppress the decomposition and obtain an accurate concentration in the urine.

For benzodiazepines, the parent compound is rarely detected in the urine, and if detected, only in trace amounts. Moreover, none of the targeted *O*-glucuronides of benzodiazepines is commercially available. Therefore, detection of the hydroxylated form of the parent compound via hydrolysis is a widely adopted practice. However, existing methods often require several hours for hydrolysis, making them unsuitable for emergency response. The method established in this study significantly reduced hydrolysis time, making it feasible for use in emergencies.

In contrast to *O*-conjugates, *N*-conjugate hydrolysis exhibited substrate specificity for specific enzymes. Enzymes, such as IMCS RT, 3S, and *E. coli*-derived enzymes demonstrated high hydrolysis efficiency under low-temperature incubations. Similarly, IMCS 3S and *E. coli*-derived enzymes achieved effective hydrolysis under high-temperature incubations. Under high-temperature incubations, fast *β*-glucuronidase achieved a hydrolysis efficiency of > 80% when reacted for over 30 min. However, it was unsuitable for the short-term hydrolysis reported in this study. Compared with these enzymes, the hydrolysis efficiency of *H. pomatia* was notably poor, and even after 360 min of reaction, the hydrolysis efficiency was less than 20%. It was presumed that the affinity of the active center of the hydrolase was different and that the substrate glucuronide could not reach to the active center successfully. Another reason for the poor hydrolysis efficiency of the *H. pomatia*-derived enzyme was the influence of contaminants in the enzyme preparation. The *H. pomatia*-derived enzyme is a crude extract solution with a low degree of purification. Its hydrolysis efficiency is reported to be lower than that of recombinant enzymes [40]. *H. pomatia*-derived enzyme poses disadvantages in the process of extracting the product using solid-phase extraction (SPE), although this enzyme is inexpensive and easy to use. As mentioned above, the *H. pomatia*-derived enzyme contains insoluble materials because it is a crude product solution and the column may become clogged during SPE extraction. To prevent this, centrifugation before SPE is indispensable, which hinders high-throughput analysis. For fast *β*-glucuronidase, reaction efficiency increased with increasing reaction temperature. The increase in the reaction temperature may have increased the hydrolysis efficiency as a result of increased the contact between the enzyme and substrate owing to the increased molecular movement of the enzyme and substrate, but the details of this improvement remain unclear.

Recombinant *β*-glucuronidase offers the advantage of a consistent quality. The IMCSzyme buffer provided by the manufacturer eliminates the need to prepare the buffer solution, facilitating hydrolysis whenever necessary, regardless of the number of samples to be tested. However, the recombinant enzyme is supplied as an aqueous solution with a set expiration date, limiting its storage over one year. *E. coli*-derived enzymes can be stored frozen for over a year; however, enzyme solutions and buffers must be prepared whenever necessary. These advantages and disadvantages must be considered along with user decisions. The results of this study suggest that pretreatment involving enzymatic hydrolysis can be performed more readily and is expected to contribute to high-throughput screening.

## Conclusion

We examined the differences in the hydrolysis efficiencies against *O*-glucuronide and *N*-glucuronide by two commercially available *β*-glucuronidases from different origins and three commercially available recombinant *β*-glucuronidases. No significant difference was observed in the hydrolysis against *O*-glucuronide depending on the enzyme used. However, differences in hydrolysis efficiencies were observed depending on the enzymes used for the hydrolysis of *N*-glucuronide. To enable high-throughput analysis of drug metabolites in human urine, the use of IMCSzyme 3S or *β*-glucuronidase derived from *E. coli* is recommended.

## Supplementary Information

Below is the link to the electronic supplementary material.

Supplementary file1 (DOCX 15 KB)
