# Supplementary material for: Investigation of commercially available recombinant and conventional β-glucuronidases to evaluate the hydrolysis efficiencies against O-glucuronides and N-glucuronides in urinary drug screening
Source: Forensic Toxicol. 2025 Mar 5;43(2):356–64. doi: 10.1007/s11419-025-00715-6 (PMC12241241; doi:10.1007/s11419-025-00715-6)
Supplement: Supplementary file 1 — Supplementary file1 (DOCX 15 KB) [file 11419_2025_715_MOESM1_ESM.docx]

***Supplementary information to the paper entitled:***

**Investigation of commercially available recombinant and conventional β-glucuronidases to evaluate the hydrolysis efficiencies against O-glucuronides and N-glucuronides in urinary drug screening**

Akira Namera^1^, Takeshi Saito^2^, Masataka Nagao^1^

^1^ Department of Forensic Medicine, Graduate School of Biomedical and Health Sciences, Hiroshima University, Hiroshima, Japan

^2^ Department of Emergency and Critical Care Medicine, Tokai University School of Medicine, Kanagawa, Japan

Table S1 Multiple reaction monitoring conditions for three target drugs in urine by liquid chromatography – tandem mass spectrometry

|  | Precursor ion (m/z) | Product ion (m/z) | Fragmentor (V) | Collision energy (eV) |
| --- | --- | --- | --- | --- |
| Amitriptyline | 278.2 | **91.2**  105.1, 117.1 | 130 | 25  25, 21 |
| Diphenhydramine | 256.2 | **107.1**  165.1, 152.1 | 90 | 9  45, 41 |
| Oxazepam | 287.1 | **240.9**  268.2, 104.1 | 130 | 21  13, 41 |
| Diazepam-*d_5_* | 290.1 | **198.0** | 130 | 33 |

Quantifier ions in bold
